# Multum in Parvo

**Published:** 1884-03

**Authors:** J. Smith Dodge

**Affiliations:** New York


### MULTUM IN PARVO.

BY J. SMITH DODGE, JR., M. D., NEW YORK.

I was glad to show my pupil the other day one of those little things which seems too small to tell of, but which is much too large to neglect. It occurred to me that it might, after all, be worth while to jot it down. A lady complained of serious pain among the right lower grinders, whenever she ate anything sweet. She could not select any tooth as the seat of it. I found a magnificent set of teeth, regular in form and position, with a few very medium-sized fillings, and one gap where the right lower first bicuspid had been. Although she was about twenty-three, the wisdom teeth had not all erupted. At the point of trouble stood a group of three teeth; second bicuspid, and first and second molars. They were finely formed, and none had ever been filled. The proximal surfaces were easily examined, and were perfect. The necks were fully covered by the gums, and no sensitive spot could be found. The grinding surfaces seemed sound, clear, and well covered with enamel, and a probe run in the usual way along the fissures discovered no defect. And yet one or the other of these three teeth was very sensitive to sweets.

Suspecting what the trouble was, since everything else was now excluded, I selected the first molar, of which the grinding surface was strongly ridged and grooved, and applied the edge of my thinnest hatchet excavator along the grooves as an explorer. No impression was made at first, but as I went to and fro, insisting that it must enter somewhere, it began to stick a little at one innocent-looking spot, and finally it was possible to enter very slightly into the fissure. A bur of the size 00 in Hodge's gauge opened a very little excavation, scarcely larger than the bur, and barely sensitive. But this I filled, with confidence that it had made all the trouble, and the next day the lady reported that sweets had lost their sting.

Of course the existence of such a minute crevice is not mentioned as anything remarkable. It is the necessary beginning of most fissure cavities, and dentists might find several any day. But the peculiarity is, that once in a great while such a crevice, too small to be discerned by any usual probing, is so exquisitely sensitive that relief must be had; and yet I never saw an instance in which the patient could certainly locate it. Neither do these cases occur in the soft teeth, which are always sensitive. These decay freely, but do not grumble till a manifest hole is formed. I have always found them in particularly good and wholesome-looking teeth. It would be interesting to know just what peculiarity of structure in these rare cases gives rise to so great a sensibility with so little exposure.

The practical upshot of it is, that when pain from sweets is complained of in a specially strong, sound tooth, and all the usual causes are sought in vain, the seat of trouble is almost certainly a microscopic defect in one of the grooves of the grinding surface, and just as likely as not at the very spot which looks most sound and perfect to ordinary examination. It seems almost like magic, that so small a filling as is needed should cure so large a pain as that which the patient complains of. But therein lies the dentist's triumph.

